# Notes and News

**Published:** 1904-11-05

**Authors:** 


### NOTES AND NEWS.

The King has conferred the title "Royal" upon the Sanitary Institute, which in the future will be known as the Royal Sanitary Institute.

A new operating theatre and extension of the out-patients' department have been opened at the Stanley Hospital, Liverpool, by the Countess of Derby. An anonymous donor presented the operating theatre, and another gentleman provided the extension. Lord and Lady Derby presented £500 to the hospital at the opening ceremony.

The medical man on board the hospital ship of the Mission to Deep Sea Fishermen has sent a letter describing the scene on board the trawlers in the North Sea, and in the hospital afterwards. He says:—"I had them all removed on board our ship. With all these wounded men on board, our floating hospital looked like a veritable battlefield. Indeed, it presented a most pathetic sight. It kept me busy with knife and needle the whole of that day, and it was not until late in the night that I had the satisfaction of seeing them all safe and snug in their cots, as far as circumstances allowed. But of the six patients three have been allowed to return to their homes, as they were progressing satisfactorily. Of the remaining three, one is quite out of danger, but the other two being very serious cases, I am still very anxious about them, and, circumstances permitting, I shall have to send them on to London if they do not progress favourably." One of the wounded fishermen is now in the London Hospital. There is very little doubt that the Russian Government will make compensation to an extent which will place the unfortunate families of the dead fishers beyond want. But the public sympathy which is so natural, can find useful vent by contributing to the funds of the excellent mission whose hospital ship rendered such good service to the wounded men.

A dispute over the will of the late Miss Frances Power Cobbe has been brought into the Lancashire Chancery Court. The Vice-Chancellor, after listening to counsel, made an order for the payment in full of a legacy of £5,000 to the British Union for the Abolition of Vivisection. The estate realised a smaller value than anticipated by Miss Cobbe, and in order to pay the legacy to the union, other legacies have to be curtailed. But this Miss Cobbe intimated she wished to be done.

The North-Eastern Hospital for Children, which is situated in one of the poorest districts of London, is very urgently in need of further support. In 1903 the accommodation was increased to meet the calls upon it. This extension of work has caused an increase of expenditure, whilst unfortunately additional subscriptions have not been forthcoming to the required extent. The hospital is now so much in debt that, unless help is soon forthcoming, some of the much-needed wards will have to be closed.

We reported last week a scheme to establish a Home of Recovery, the object of which is to provide the London hospitals with an establishment where patients can continue lengthened treatment and secure the benefit of fresh air, and at the same time relieve the pressure on hospital accommodation. A protest has arisen with which we sympathise. It is pointed out that the scheme is on far too small a scale to be effective. Further, it is suggested that it would be much better to let each hospital make provision for cases at its convalescent home. This would necessitate the employment of a resident medical officer, and of a larger staff of nurses at each home. All these suggestions show a gradual recognition of the fact that London cases should be treated so far as possible in the country. A system of emergency hospitals in London, and the creation of greater hospital accommodation in the country is probably only a question of time.

On Tuesday Mr. John Burns, M.P., delivered a lecture at Manchester on the Drink Evil, which he declared was the chief cause of poverty, lunacy, and crime. He gave it as the opinion of Sir Andrew Clark, after an experience of 20 years at the London Hospital, that seven out of every 10 patients treated there were victims of physical injury caused by drink. He said that in 1903, 400,000 accident, casualty, and emergency cases were received in London hospitals, and he applied the moderate percentage of five as being due to drink. As the hospitals look to the railway companies and business firms in their neighbourhood to contribute to their funds in support of patients who are received from them, so we are tempted to suggest that if brewers, distillers, and licensed victuallers were to contribute to the hospitals, to the modest extent of five per cent. of their profits (being in the same proportion as the number of cases which receive hospital relief, through drink) the long-standing difficulty in raising voluntary support would be removed.

